# Single nucleotide polymorphisms rs148582811 regulates its host gene *ARVCF* expression to affect nicotine-associated hippocampus-dependent memory

**DOI:** 10.1016/j.isci.2023.108335

**Published:** 2023-10-28

**Authors:** Zhongli Yang, Jiali Chen, Haijun Han, Yan Wang, Xiaoqiang Shi, Bin Zhang, Ying Mao, Andria N. Li, Wenji Yuan, Jianhua Yao, Ming D. Li

**Affiliations:** 1 State Key Laboratory for Diagnosis and Treatment of Infectious Diseases, National Clinical Research Center for Infectious Diseases, National Medical Center for Infectious Diseases, Collaborative Innovation Center for Diagnosis and Treatment of Infectious Diseases, The First Affiliated Hospital, Zhejiang University School of Medicine, Hangzhou 310009, China; 2 Joint Institute of Smoking and Health, Kunming, Yunnan 650024, China; 3 Vanderbilt University School of Medicine, Nashville, TN 37240, USA; 4 Research Center for Air Pollution and Health, Zhejiang University, Hangzhou 310058, China

**Keywords:** Natural sciences, Biological sciences, Neuroscience, Behavioral neuroscience, Clinical neuroscience

## Abstract

Although numerous susceptibility loci are nominated for nicotine dependence (ND), no report showed any association of *ARVCF* with ND. Through genome-wide sequencing analysis, we first identified genetic variants associated nominally with ND and then replicated them in an independent sample. Of the six replicated variants, rs148582811 in *ARVCF* located in the enhancer-associated marker peak is attractive. The effective-median-based Mendelian randomization analysis indicated that *ARVCF* is a causal gene for ND. RNA-seq analysis detected decreased *ARVCF* expression in smokers compared to nonsmokers. Luciferase reporter assays indicated that rs148582811 and its located DNA fragment allele-specifically regulated *ARVCF* expression. Immunoprecipitation analysis revealed that transcription factor X-ray repair cross-complementing protein 5 (XRCC5) bound to the DNA fragment containing rs148582811 and allele-specifically regulated *ARVCF* expression at the mRNA and protein levels. With the *Arvcf* knockout mouse model, we showed that *Arvcf* deletion not only impairs hippocampus-dependent learning and memory, but also alleviated nicotine-induced memory deficits.

## Highlights

• SNP rs148582811 in *ARVCF* associated significantly with ND is identified
• The EMIC analysis reveals that *ARVCF* is a causal gene for ND
• XRCC5 binding to SNP rs148582811 regulates *ARVCF* expression
• *Arvcf* plays a key role in nicotine-associated hippocampus-dependent memory

## Graphical abstract

**Figure:**
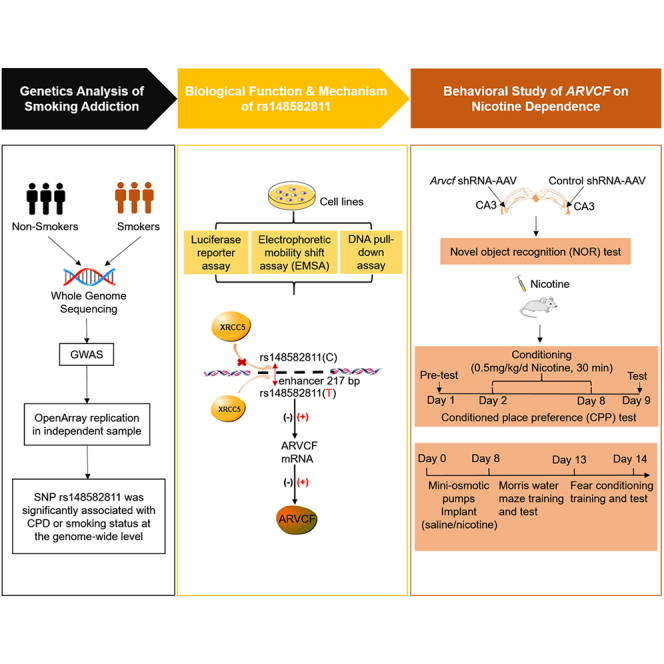

## Introduction

Tobacco use remains a high rate worldwide, notably in China.^1^ Despite the known healthy risks and strict legislation against it, the prevalence of smoking in China increased 2-fold in 2016 compared with 1985.^2^ With an increased tobacco use in China, it was predicted that the death toll could rise to 3 million per year by 2050.^3^ Thus, further research on the pathogenesis of nicotine dependence (ND) will promote the development of effective treatment strategies for the disease.

ND plays a crucial role in maintaining smoking-related behaviors.^4^ Numerous epidemiologic studies have provided strong evidence that genetics affects greatly on the development of ND, with an average heritability of 56%.^5^ Therefore, identifying genetic variants and biological function underlying ND are critical for treating those addicted tobacco smokers.

Although a great number of susceptibility loci for ND have been identified during the past years,^6^^,^^7^ most of them are synonymous variants and difficulty to explain their involvement in the pathogenesis of ND at the molecular level. Even for a few of those identified nonsynonymous variants, the biological functions and regulatory mechanisms underlying their involvement in the pathogenesis of ND have rarely investigated. One of the most extensively investigated variants was SNP rs16969968 in exon 5 of neuronal nicotinic acetylcholine receptor (nAChR) α5 subunit gene (*CHRNA5*), which changes an aspartic acid residue into asparagine at position 398 (D398N) of the α5 subunit protein sequence and is tightly linked to SNP rs1051730 in *CHRNA3*; both of them are highly associated with ND.^8^^,^^9^^,^^10^^,^^11^^,^^12^^,^^13^

On the other hand, several recent studies have revealed that low-frequency and even rare coding variants can play a significant role in the involvement of each susceptibility gene for ND.^14^^,^^15^^,^^16^ In some cases, their contribution to a disease could be much greater than those observed in common variants.^17^ Thus, it has been suggested that the whole genome sequencing (WGS) analysis holds great potential for finding rare variants with large biological effects, which would greatly increase tractability for biological experiments and reveals novel susceptibility genes as well as biological pathways underlying ND.

Association of ND with cognitive deficits has been documented in both preclinical animal^18^^,^^19^ and human studies.^20^^,^^21^^,^^22^^,^^23^ Relative to healthy controls, it was found that smokers have cognitive deficits in auditory-verbal and visuospatial learning, visuospatial memory, cognitive efficiency, executive skills, and processing speed.^20^ In a population-based study of 2,163 participants, smokers were found to have deficits in attention, working memory, and impulse control functions.^23^ There is increasing evidence suggesting that nicotine has direct effect in the hippocampus altering learning and memory, and these changes in cognitive function may impact susceptibility to develop and maintain ND.^24^^,^^25^ Additionally, preclinical studies further indicated that nAChR subunits, especially α7 and β2 subunits, play important roles in the cognitive effects of nicotine.^26^^,^^27^ Although information at different levels of analysis are continuing to emerge, the neurobiological mechanisms underlying nicotine’s effects on cognitive function remain to be further elucidated.

Specifically, this study had the following two main objectives: 1) to discover more variants, especially those rare ones, from less studied ethnic populations on smoking such as Chinese smokers through WGS analysis; and 2) to select one representative rare variant and the gene where it locates to demonstrate their biological function and molecular mechanism underlying smoking-associated phenotypes by using both *in vivo* and *in vitro* approaches.

## Results

### Single nucleotide polymorphisms rs148582811 located in *ARVCF* was significantly associated with tobacco smoking

Our WGS analysis of 1,329 Chinese male subjects consisting of 805 heavy smokers and 524 age-matched non-smokers revealed several novel loci associated significantly with smoking status or CPD at a genome-wide significance (p < 5 × 10^−8^; Figure S1). A detailed description of these subjects is given in Table 1. For these putative susceptibility loci, 31 SNPs of potential interest based on their predicted biological functions were further genotyped using *TaqMan* OpenArray in 3,726 independent samples (Figure S2 and Table S3), and six of them were successfully replicated (Table 2).

**Table 1:** Characteristics of clinical samples used in the study

Characteristics		WGS	Replication
No. of Subjects	Smokers	805	1,811
	Non-smokers	524	1,915
Age (Years; Mean ± S.D.)	Smokers	40.4 (±8.9)	40.5 (±10.0)
	Non-smokers	40.9 (±7.2)	35.9 (±11.5)
CPD (Mean ± S.D.)	Smokers	20.3 (±6.2)	16.4 (±8.7)
	Non-smokers	NA	NA

**Table 2:** A list of six variants replicated in an independent sample with 3,744 subjects

Gene	SNP ID	Chr.	Genomic Position	Minor allele	WGS Samples (N = 1,329)			Replication Samples (N = 3,744)			P_Meta_	Smoking Phenotype
					MAF	OR/ Beta	p Value	MAF	OR/ Beta	p Value		
NA	rs796305983	2	92248401	G	0.053	0.27	2.19 × 10^−11^	0.076	0.71	3.30 × 10^−5^	1.62 × 10^−12^	SS
*CTBP2*	rs3208623	10	126677501	A	0.07	0.39	3.19 × 10^−8^	0.087	0.65	2.88 × 10^−6^	6.82 × 10^−12^	SS
*FOXP1*	rs7635815	3	70886939	C	0.023	5.32	4.86 × 10^−8^	0.12	1.94	2.22 × 10^−5^	7.02 × 10^−12^	CPD
*RP11-260A9.6*	rs9674567	17	25298140	A	0.036	0.26	1.21 × 10^−8^	0.051	0.60	1.01 × 10^−5^	1.35 × 10^−11^	SS
*DGCR6, PRODH*	rs796774020	22	18880657	T	0.101	0.38	2.74 × 10^−11^	0.09	0.79	3.60 × 10^−3^	3.20 × 10^−9^	SS
*COMT, ARVCF*	rs148582811	22	19977012	T	0.012	7.82	1.18 × 10^−7^	0.041	1.31	2.87 × 10^−2^	1.21 × 10^−7^	CPD

Chr. = Chromosome, MAF = Minor Allele Frequency, OR = Odds Ratio, NA = Not Applicable, SS = Smoking Status, CPD = Cigarettes Per Day.

**Figure 1:**
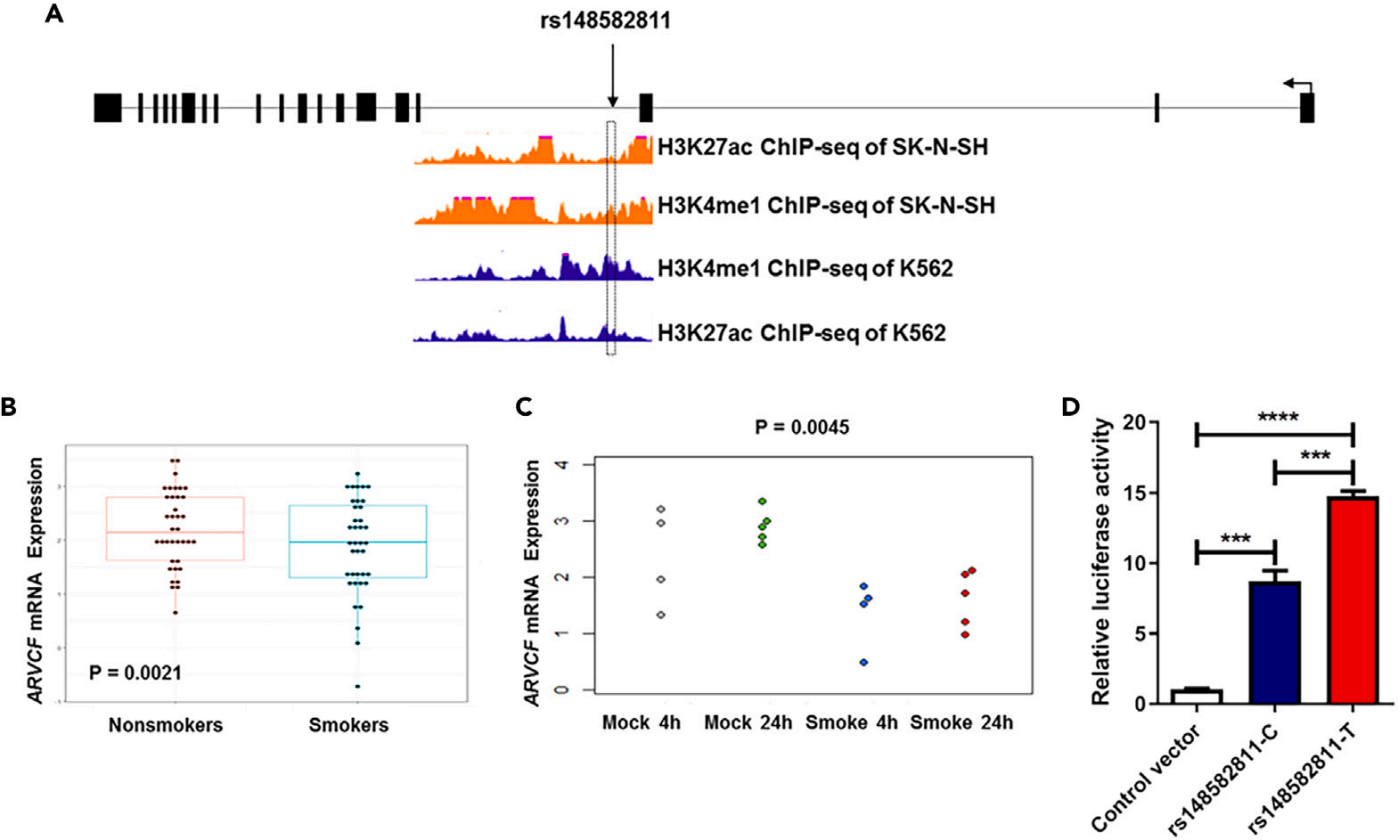
The predicted enhancer role of rs148582811 in *ARVCF* (A) Prediction of potential regulatory function of rs148582811 by using ENCODE ChIP-seq data. (B) Comparison of *ARVCF* mRNA expression between 38 smokers and 36 non-smokers. (C) *ARVCF* mRNA expression levels in normal bronchial epithelial cells exposed to cigarette smoke compared with controls (downloaded from GEO microarray dataset GDS1348). (D) Comparison of relative luciferase activity in Controls, rs148582811-C or -T allele in HEK293T cells (∗∗∗p < 0.001).

By annotating potential biological functions for these six significant SNPs using chromatin immunoprecipitation sequencing (ChIP-seq) data from the ENCODE consortium,^28^ we found that an intronic SNP rs148582811 in *ARVCF* was located in a genomic region which was enriched for the enhancer-associated H3K4me1 and H3K27ac histone marks^29^^,^^30^^,^^31^ in both SK-N-SH and K562 cells (Figure 1A), suggesting this genomic region may function as an enhancer. Further, gene-based association test using extended Simes procedure (GATES) indicated that *ARVCF* was significantly associated with ND (p = 0.00056). We also did gene-based association analysis for *ARVCF* with the same approach based on the GTEx eQTLs data, which revealed a significant association of *ARVCF* expression with ND in forebrain region (p = 0.0012).

Next, we compared RNA expression level of *ARVCF* between smokers and non-smokers in two independent datasets with one from our laboratory (Figure 1B)^32^ and the other from the GEO database (GDS1348; Figure 1C) and found that the mRNA expression of *ARVCF* was significantly lower in the smoker group compared to non-smoker group (p < 0.01). Further, we did the effective-median-based Mendelian randomization for inferring the causal genes (EMIC) approach for *ARVCF* with the GWAS results from our sample and GTEx expression data in brain, we found that *ARVCF* is a causal gene for ND in forebrain region (min EMIC p = 0.021), suggesting that *ARVCF* overexpression might increase the risk of ND (Figure S3).

### Both rs148582811 and its containing DNA region allele-specifically regulate *ARVCF* expression

To examine if the intronic DNA region containing rs148582811 acts as an enhancer, we first cloned the DNA fragment containing rs148582811-C or -T allele into a luciferase vector and then conducted luciferase reporter assay in HEK293T cells. We found that the DNA fragment containing rs148582811-T allele significantly increased the enhancer activity compared with that containing rs148582811-C allele (p < 0.001) (Figure 1D).

**Figure 2:**
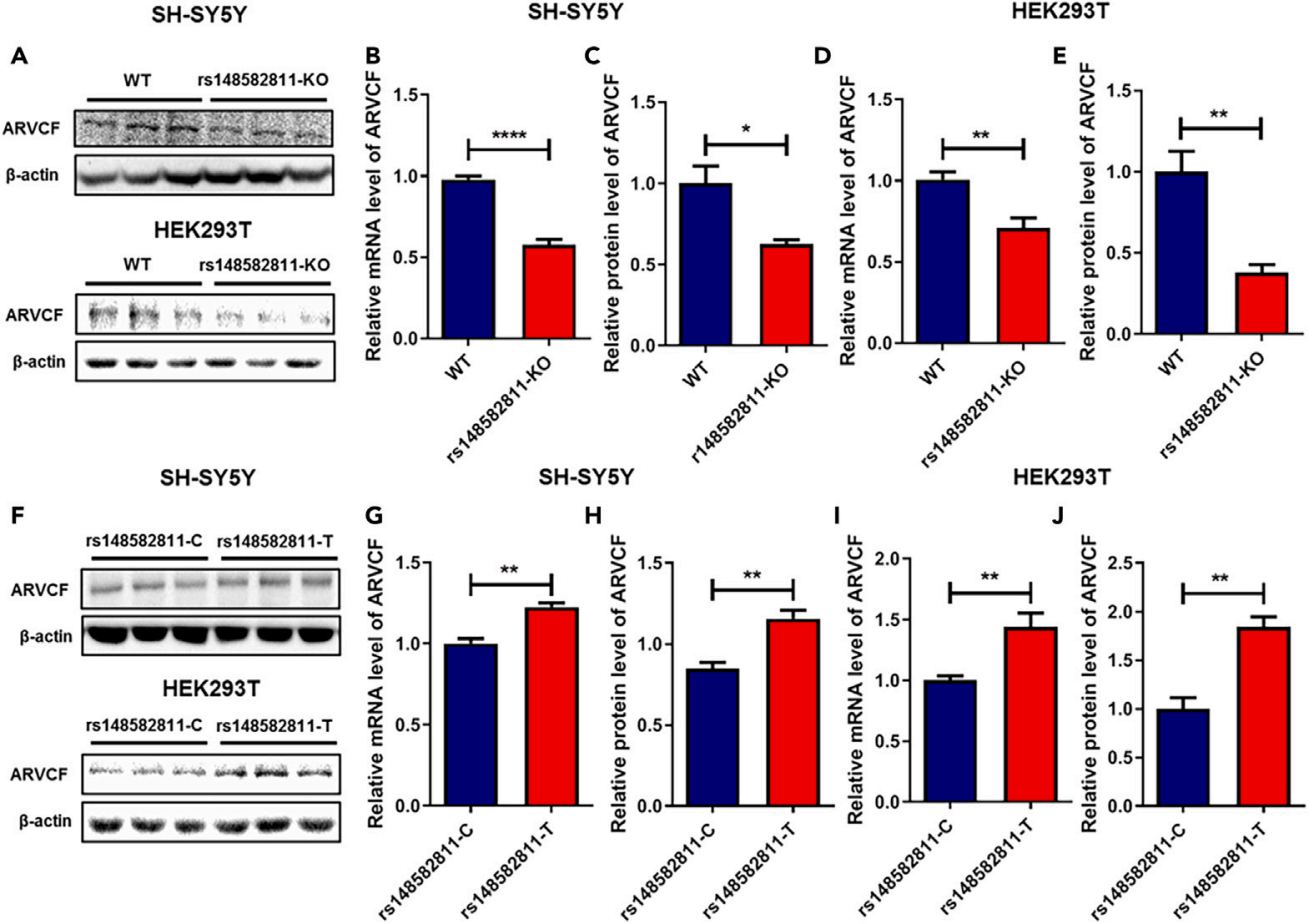
Regulatory effect of rs148582811 and its containing DNA genomic region on the expression of *ARVCF* (A and F) Representative pictures of Western blotting of *ARVCF* in rs148582811-KO and rs148582811-T SH-SY5Y or HEK293T cells. Regulated *ARVCF* mRNA and protein levels by the genomic region containing rs148582811 were examined in rs148582811-KO SH-SY5Y (B and C) and rs148582811-KO HEK293T (D and E) cells. Regulated *ARVCF* mRNA and protein expression level by rs148582811 were determined in rs148582811-T SH-SY5Y (G and H) and rs148582811-T HEK293T (I and J) cells. Values are shown as Mean ± SEM of at least three independent experiments. ∗p < 0.05; ∗∗p < 0.01; ∗∗∗∗p < 0.0001.

To determine whether rs148582811 and its containing DNA region regulate *ARVCF* expression, we used CRISPR/Cas9 to independently edit SH-SY5Y and HEK293T cells with a 217 bp fragment deletion containing rs148582811 site (Figure S4A), which were confirmed by Sanger sequencing (Figures S4B and S4C). As shown in Figures 2A–2E, significant decreases in *ARVCF* mRNA and protein expression were detected in both rs148582811-KO SH-SY5Y and HEK293T cells compared with the WT cell lines (p < 0.05). Further, we investigated the role of rs148582811 in regulating *ARVCF* expression. Both SH-SY5Y and HEK293T cells were transfected with homologous repair template including rs148582811-T allele in addition to Cas9-sgRNA plasmid (Figure S4D). And then, the cells carrying T-allele were generated and verified by Sanger sequencing (Figures S4E and S4F). Subsequently, qRT-PCR and Western blotting analyses demonstrated that *ARVCF* expression increased significantly in the rs148582811-T SH-SY5Y and HEK239T cells (p < 0.01) (Figures 2F–2J) compared with rs148582811-C cells. These results provided direct evidence that rs148582811 plays a regulatory role on *ARVCF* expression.

### X-ray repair cross-complementing protein 5 binds to rs148582811-containing DNA region

**Figure 3:**
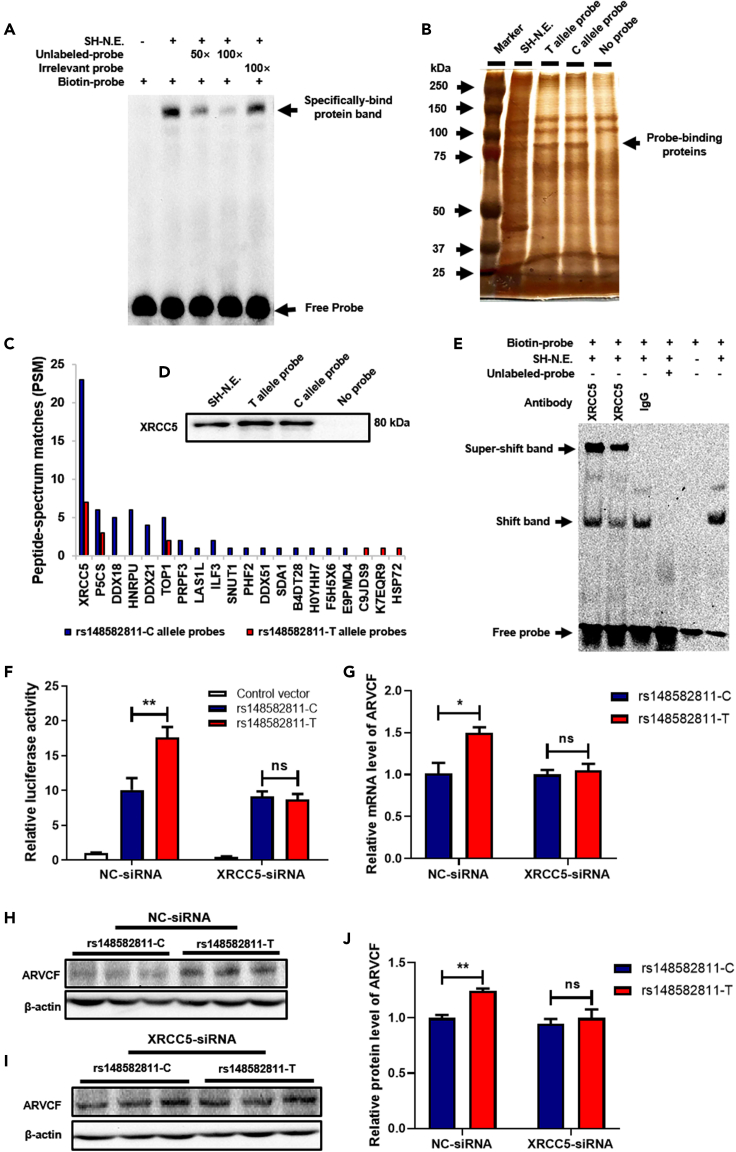
Rs148582811 allele-specifically regulates *ARVCF* expression through binding to XRCC5 (A) Electromobility shift assay (EMSA) was performed by using biotinylated double-stranded probes containing the rs148582811 incubated in nuclear extracts from SH-SY5Y with or without competitors. (B) DNA pull-down assay was conducted by separating the bounded proteins to rs148582811-containing DNA probe with 8% acrylamide SDS gel and then the gel was stained with silver. (C) The binding proteins with the size of about 90 kDa were identified by Mass Spectrometry. (D) The XRCC5 protein level identified from mass spectrometry was examined by Western blotting. (E) Super-shift EMSA was performed by incubating biotinylated double-stranded probes in nuclear extract from SH-SY5Y with or without competitors and antibody. Lane 1: probe with rs148582811-C allele, Lane 2: probe with rs148582811-T allele. (F) The luciferase reporter assay was conducted in HEK293T cells by co-transfecting with the pGL4.26 vector harboring *renilla* luciferase, either rs148582811-C or T allele, and either XRCC5-siRNA or control-siRNA. (G and J) Rs148582811-C and -T SH-SY5Y cells were transfected with negative control-siRNA (NC-siRNA) or XRCC5-siRNA, and then *ARVCF* mRNA (G) and protein (J) levels were determined at 48 h after transfection. (H and I) The representative pictures of Western blotting of *ARVCF* in rs148582811-C and rs148582811-T SH-SY5Y cells transfected with NC-siRNA (H) or XRCC5-siRNA (I). Values are shown as Mean ± SEM of at least three independent experiments. ∗p < 0.05; ∗∗p < 0.01; ns = no significance.

Next, we wanted to determine if there existed any transcriptional factor (TF) that interacts with the rs148582811-containing DNA region. By conducting EMSA through incubation nuclear extracts from SH-SY5Y cells with double-stranded DNA probes of predicted enhancer element, we found that the nuclear proteins bound to the probes leading to the formation of one predominant complex (Figure 3A). In the competition assay, the unlabeled DNA diminished the shifted band in a dose-dependent manner, but the mutant probe had no obvious effect on the band. To better understand which protein binds specifically to rs148582811-containing DNA region, DNA pull-down assay was conducted to purify the binding TFs with the DNA probes used in the EMSA assay and nuclear extracts from SH-SY5Y cells. The silver staining of proteins recovered from pull-down experiments showed proteins with a molecular weight of around 90 kDa bound to rs148582811-C and -T probes but not to the control probe (Figure 3B). Among these ∼90 kDa proteins, XRCC5 was firstly identified by mass spectrometry (Figure 3C) and then validated by Western blotting (Figure 3D). To further confirm that XRCC5 indeed binds to rs148582811-containing DNA region, we performed super-shift EMSA by using anti-XRCC5 antibody as well as probes for both C and T alleles of rs148582811. As a result, when the C and T probes were incubated with the anti-XRCC5 antibody, the shifted band was markedly diminished along with the formation of a super-shifted band (Figure 3E, Lanes 1 and 2). Together, these results indicated that the rs148582811-containing DNA region indeed interacts with XRCC5.

### Rs148582811 allele-specifically modulates *ARVCF* expression by binding to X-ray repair cross-complementing protein 5

Next, we determined whether XRCC5 executed its regulatory effect on *ARVCF* expression through binding to the rs148582811-C or -T allele. By the co-transfection of luciferase reporter constructs containing rs148582811-C or -T allele with either XRCC5-siRNA or control-siRNA, we found that the cells transfected with T allele significantly increased luciferase activity compared to the C allele in co-transfected with control-siRNA group (p < 0.01) (Figure 3F, left), which was consistent with the finding when the luciferase construct was transfected alone. However, in co-transfected with XRCC5-siRNA group, the luciferase activity showed no significant difference between rs148582811-C and T alleles (Figure 3F, right). To further investigate whether the regulation of rs148582811 on *ARVCF* expression was modulated by XRCC5, XRCC5-siRNA was used to knockdown its expression. We found that there were significant differences between rs148582811-C and rs148582811-T cells in control-siRNA group, however, the downregulation of XRCC5 in rs148582811-C and rs148582811-T SH-SY5Y cells did not show allele-specific regulation on *ARVCF* (Figures 3G–3J). Together, these findings support our hypothesis that XRCC5 plays an important role in the regulatory effect of rs148582811 on *ARVCF* expression by binding to both alleles of the SNP.

### *ARVCF* impacts hippocampus-dependent memory in mice

**Figure 4:**
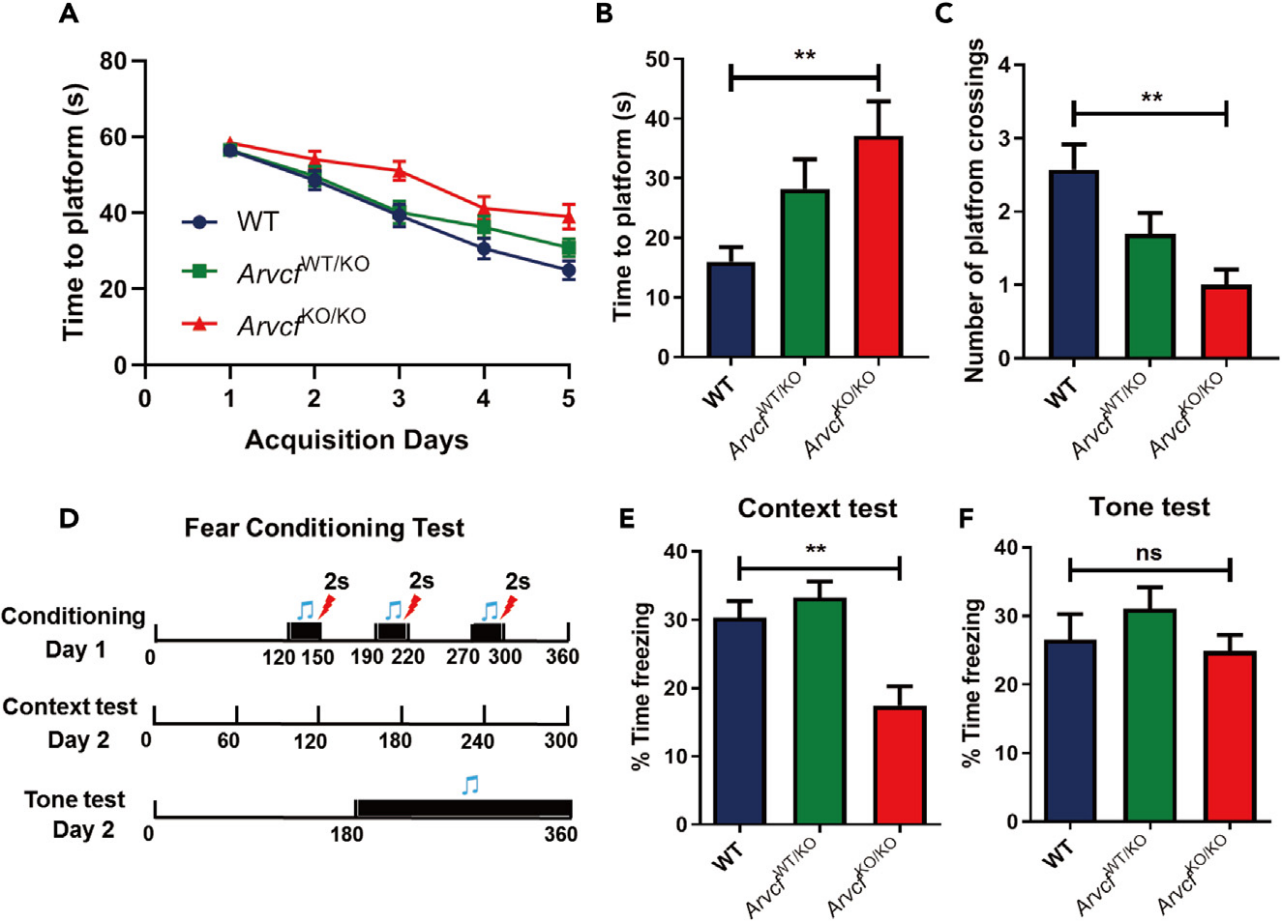
Determining the effect of *ARVCF* on hippocampal-dependent learning and memory in *Arvcf* KO mice by using Morris water maze (MWM) test and fear conditioning test (A) The reduced time to platform during a 5-day training phase. (B) The time to platform and (C) number of platform crossings during probe test on the day 6 in wild-type (WT), *Arvcf*^WT/KO^, and *Arvcf*^KO/KO^ groups. ∗∗ p < 0.01. (D) Schematic of fear conditioning test. On the day 1, which is a conditioning test, three pairs of tone (30 s, 5 kHz, 85 dB) and foot shock (0.8 mA, 2 s) were presented. On the day 2, which is context test followed by tone test after 2 h, no tone and foot shock were presented in the context test, and altered apparatus but only tone (30 s, 5 kHz, 85 dB) presented in the tone test. (E and F) The percentage of freezing time during context test (E) and tone test (F) in WT, *Arvcf*^WT/KO^, and *Arvcf*^KO/KO^ groups. ∗∗ p < 0.01.

As a member of p120 catenin family linked to the synapse reorganization and memory, we were interested to determine whether *ARVCF* has any role in learning and memory. By using CRISPR/Cas9 system, we generated the *Arvcf*-KO mouse model with a 5805bp deletion (Figures S4G and S4H) and used them to test the effects of *Arvcf* on learning and memory by comparing *Arvcf* ^WT/KO^ and *Arvcf* ^KO/KO^ mice with WT mice. In MWM test, the time to platform was significantly enlonged on day 6 in *Arvcf* ^KO/KO^ mice compared with that in the WT mice (p < 0.01) (Figure 4B). Moreover, the platform crossing times were significantly less in *Arvcf* ^KO/KO^ mice compared with those in the WT mice (p < 0.01) (Figure 4C).

We then assessed the contextual and cued fear memory in *Arvcf*-KO mice (Figure 4D). In the contextual fear memory test, *Arvcf* ^KO/KO^ mice were frozen at significantly lower level than the WT mice (p < 0.01) (Figure 4E). However, the results of cued (tone) fear memory test showed no obvious difference of freezing between the WT and *Arvcf* ^KO/KO^ mice (Figure 4F). No significant change of memory was observed between the WT and *Arvcf* ^WT/KO^ mice in both MWM and fear conditioning experiments. Moreover, the significant change of contextual memory but not cued memory indicated that *Arvcf*-KO led to the hippocampus-dependent memory deficient.

**Figure 5:**
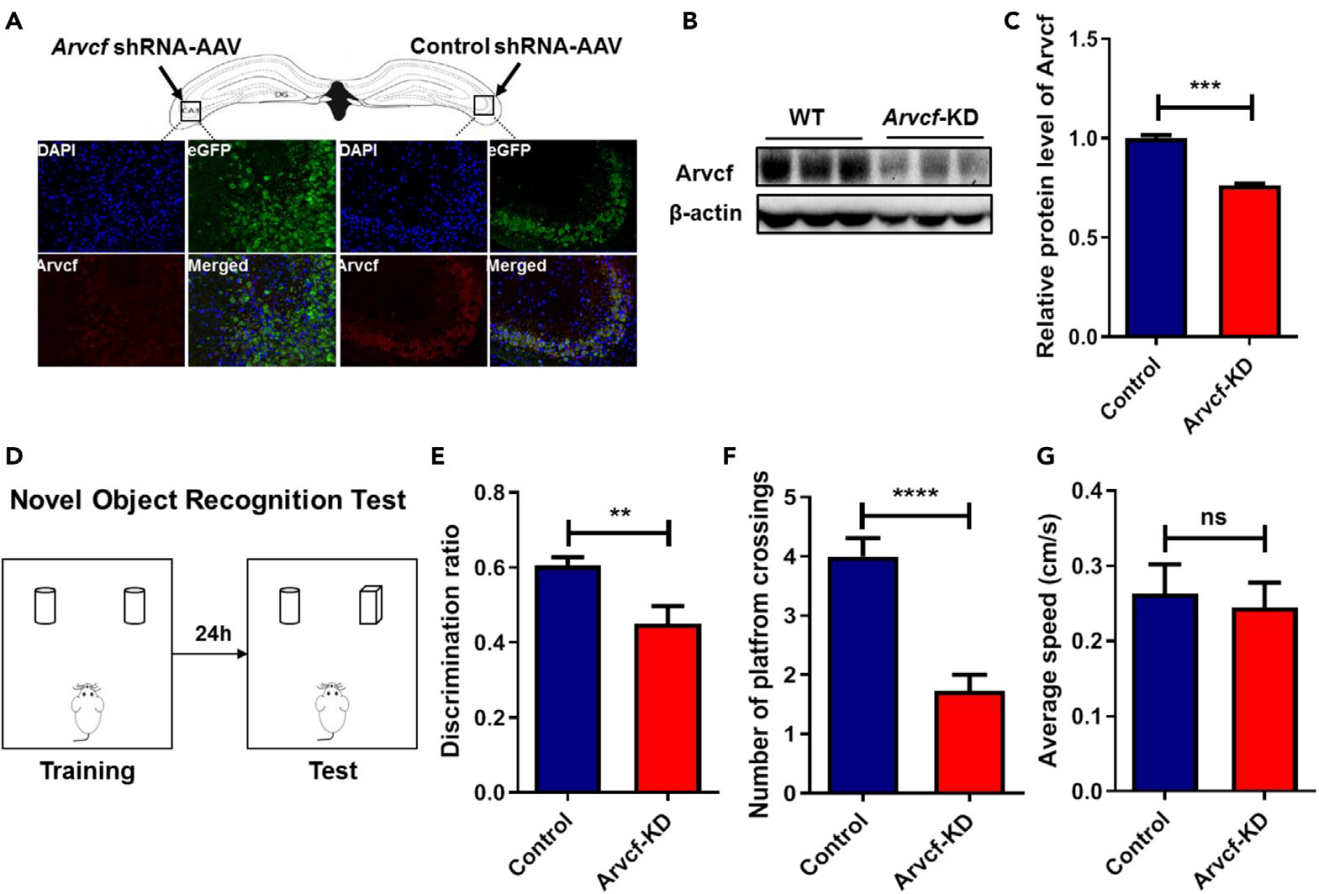
Determining the effect of *ARVCF* on hippocampal-dependent learning and memory in mouse hippocampal CA3 region by using Adeno-associated virus system *Arvcf* was knocked down (KD) by shRNA-AAV in the CA3 brain region of mouse hippocampus, then fluorescence immunostaining (A) and Western blotting (B and C) were used to detect the expression of *ARVCF*. (D) Schematic of novel object recognition (NOR) test. During the training phase, each mouse was subjected to two identical objects for 8 min, and 24-h later, one of the objects was replaced with a novel object and all mice explored for 5 min. (E) The discrimination ratio of novel object preference in control and *Arvcf*-KD groups. (F) The number of platform crossings and (G) average speed changes in MWM test after *Arvcf* knockdown. N = 10–14 per experimental group for *Arvcf*-KO study, and n = 8–12 per experimental group for *Arvcf*-KD study. Values are shown as Mean ± SEM. ∗p < 0.05; ∗∗p < 0.01; ∗∗∗p < 0.001; ∗∗∗∗p < 0.0001; ns = no significance.

To further investigate the role of *ARVCF* on hippocampus-dependent learning and memory, Adeno-associated virus (AAV) system, harboring an *Arvcf*-shRNA, was used to knockdown *Arvcf* expression in mouse hippocampal CA3 subregion, an important brain region in learning and memory.^27^ Both immunofluorescent staining and Western blotting analysis showed a significant reduction of Arvcf protein expression in mouse hippocampal CA3 (p < 0.001) (Figures 5A–5C). Then, we assessed the changes of object and spatial memory of *Arvcf*-KD mice by using novel object recognition (Figure 5D) and MWM experiments (Figures 5E–5G). Compared with the control group, *Arvcf*-KD mice showed reduced preference toward novel object (p < 0.01; Figure 5E). In MWM test, the number of platform crossings was less in *Arvcf*-KD group compared with that in the control group (p < 0.0001) (Figure 5F), and no difference was detected in the average swimming speed between the two groups (Figure 5G).

### *ARVCF* plays a vital role in the rewarding effect of nicotine and nicotine-associated memory

**Figure 6:**
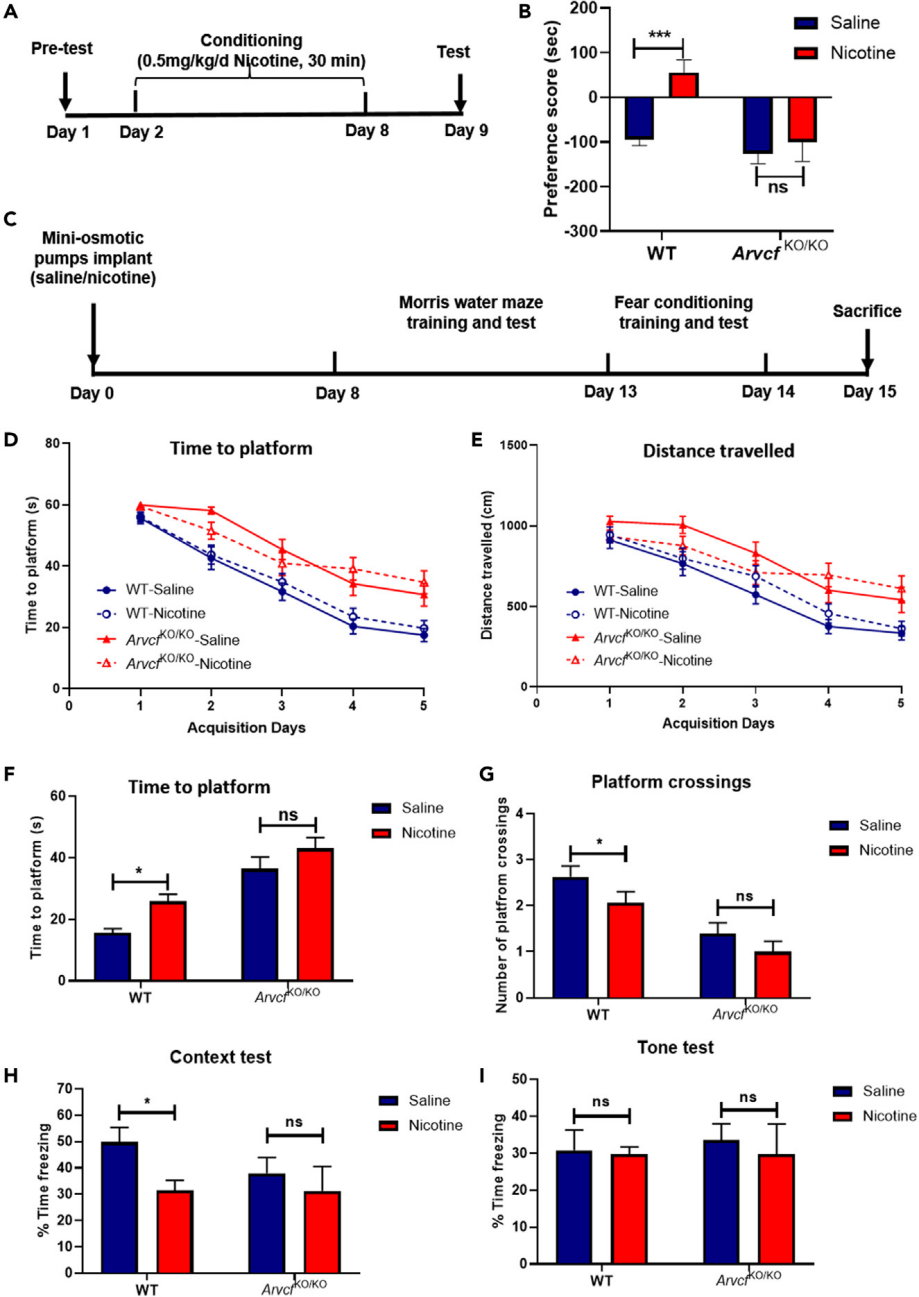
Determining the involvement of *ARVCF* in chronic nicotine-induced impairment of memory (A) Experimental design of conditioned place preference (CPP) test. (B) Comparison of rewarding effects of nicotine in wild-type (WT) and *Arvcf*^KO/KO^ mice with the CPP paradigm. (C) Schematic of chronic nicotine treatment with mini-osmotic pump on WT and *Arvcf*^KO/KO^ mice followed with MWM test and fear conditioning test. (D) The reduced time to platform and (E) distance traveled to platform during a 5-day training phase. (F) The time to platform and (G) number of platform crossings during probe test on day 13 after chronic nicotine treatment. The percentage of freezing time during (H) context test and (I) tone test in WT and *Arvcf*^KO/KO^ mice after chronic nicotine treatment. Values are shown as Mean ± SEM. ∗p < 0.05; ∗∗p < 0.01; ∗∗∗p < 0.001; ns = no significance.

To assess the biological role of *ARVCF* on the rewarding effect of nicotine, we performed conditional place preference (CPP) assay in *Arvcf*-KO mice and corresponding WT mice. Both WT and *Arvcf* ^KO/KO^ mice were conditioned to nicotine treatment in a 7-day unbiased CPP paradigm (Figure 6A), and were compared with the saline-control mice. On the day 9 of treatment (Figure 6B), CPP test showed a robust preference for the nicotine-paired compartment in WT group (p = 0.00027), whereas *Arvcf* ^KO/KO^ mice did not show an obvious preference for nicotine treatment (p > 0.05). These results demonstrated that *Arvcf* plays a role in mediating the rewarding effect of nicotine on mice.

As nicotine is known to execute its effects on hippocampus-dependent learning and memory,^27^^,^^33^ we then determined whether *Arvcf* was involved in the effects of chronic nicotine treatment on nicotine-associated memory in WT and *Arvcf* ^KO/KO^ mice (Figure 6C). In MWM test, during the training phase on days 8–12 after pump implantation (Figures 6D and 6E), although the time to platform and distance traveled to platform were reduced in all four groups day by day, the *Arvcf* ^KO/KO^ mice spent significantly more time and traveled more distances to reach the platform. During the test phase on day 13, compared with WT mice in the saline group, the WT mice in the nicotine-treated group spent significantly greater time reaching the location of platform (p < 0.05; Figure 6F) and crossed less numbers to the platform (p < 0.05; Figure 6G). However, no significant differences were observed in *Arvcf* ^KO/KO^ mice between the saline and nicotine group. Together, these results indicated that *Arvcf* knockout attenuated chronic nicotine-induced spatial memory impairment.

Further, in the fear conditioning test during the days 13 and 14 of nicotine or saline treatment, we examined whether *Arvcf* knockout could alter nicotine-associated fear memory. As shown in Figures 6H and 6I, in WT mice, chronic nicotine treatment led to significantly reduction of freezing time during contextual fear response test (p < 0.05), but there was no effect on cued fear response. In contrast, in *Arvcf* ^KO/KO^ mice, chronic nicotine treatment led to no significant change in freezing time during both contextual (Figure 6H, right) and cued fear responses (Figure 6I, right), suggesting that *Arvcf* deletion significantly attenuated chronic nicotine-induced deficits in hippocampus-dependent fear memory. Together, these findings indicated that *Arvcf* plays a key role in chronic nicotine-associated hippocampus-dependent memory deficient.

## Discussion

Up to now, the identified susceptible variants for ND by genetic association analysis could only explain a small portion of the estimated heritability for ND. To find more causal variants, we conducted WGS analysis on 1,329 Chinese samples and found multiple SNPs associated significantly with smoking at the genomic level. Of them, six were replicated in 3,726 independent subjects, indicating they were significantly associated with smoking. Since identified SNPs by GWAS are mostly enriched in DNA regulatory elements and can regulate gene expression,^34^ we then used ChIP-seq data from ENCODE database to predict the biological function of the identified variants. This revealed that SNP rs148582811 in *ARVCF* gene overlapped with the enhancer marker signals (H3K4me1 and H3K27ac) in both SK-N-SH and K563 cells, suggesting the potential regulatory function of rs148582811. Further, our gene-based association test demonstrated that *ARVCF* was significantly associated with ND at both the GWAS results from our sample and the eQTL results in forebrain region of GTEx dataset.^35^ In addition, by using the RNA-seq data from our laboratory^32^ and public GEO dataset, we found that *ARVCF* mRNA expression level was significantly lower in smokers compared to non-smokers. Finally, our EMIC analysis revealed that *ARVCF* is a causal gene for smoking addiction where we found an increased *ARVCF* expression can lead to an increased risk of ND.

During the past years, although a number of susceptibility genes and variants have been identified for ND, whether these genetic findings could be applied to clinic practices depends largely on their biological functions.^36^ By using luciferase reporter assay to initially explore the biological function of rs148582811, we found that rs148582811 not only acted as an enhancer, but also discovered that rs148582811-C or T allele had different regulatory functions, which formed the major reason for us to focus on the functional study of this rare variant in the present report.

To determine whether the susceptibility variant rs148582811 has any biological function, CRISPR/Cas9 gene editing technique is commonly used, which can accurately edit the target site or region on the genome and provide effective experimental models for studying functional mechanisms for the variants of interest.^37^ However, due to the low efficiency of single-base editing of CRISPR/Cas9 system, there were very limited reports regarding the application of CRISPR/Cas9 technology in SNP functional research.^38^^,^^39^ Based on the above genetic and functional evidence, we hypothesized that the SNP rs148582811 could regulate *ARVCF* expression in an allele-specific manner. By applying CRISPR/Cas9 gene editing technique, the locus of rs148582811 and the region where it locates were deleted. In the rs148582811-KO SH-SY5Y and HEK293T cells, both *ARVCF* mRNA and protein expression were significantly decreased. Furthermore, in the SH-SY5Y and HEK293T cells carrying rs148582811-T allele, both *ARVCF* mRNA and protein expression levels were significantly higher than those in rs148582811-C cells. These findings demonstrated that the rs148582811 alleles affected the expression of *ARVCF* by regulating the activity of enhancer element.

Given the potential important biological function of rs148582811, we also explored the mechanism underlying the function of rs148582811. Considering that the nuclear protein generally regulates gene expression through binding to the regulatory region of the genome,^40^ we conducted the EMSA, DNA pull-down and protein mass spectrometry experiments, which revealed that nuclear protein XRCC5 bound to the rs148582811-containing DNA region. XRCC5, also known as Ku80, is a component of the DNA-dependent protein kinase, existing as a heterodimer with XRCC6.^41^^,^^42^ Because XRCC5 binds to DNA and was reported to function as a transcription factor,^43^^,^^44^^,^^45^ we then performed rs148582811 luciferase report experiment in XRCC5-KD HEK293T cells, and observed no significant difference of enhancer activity between rs148582811-C and -T cells. Further, we found that the significant difference of *ARVCF* expression between the rs148582811-C and rs148582811-T SH-SY5Y cells were completely abolished after XRCC5 knockdown, indicating that XRCC5 is able to regulate the functional difference between C and T alleles of rs148582811.

The protein encoded by *ARVCF* belongs to p120ctn catenin family, which plays a significant functional role in various aspects of neuronal morphogenesis, neurodevelopmental and neurological disorders.^46^ Recent works have confirmed that *ARVCF* is involved in the modulation of cell-cell adhesion^47^^,^^48^ and essential to many developmental processes including neuronal rearrangement and migration.^49^^,^^50^ *ARVCF* has also been shown to play a critical function in regulating cadherin level,^51^ while N-cadherin was reported to promote synaptic differentiation and regulate the proliferation and differentiation of ventral midbrain dopaminergic progenitor.^52^^,^^53^^,^^54^ In addition, it has been reported that *ARVCF* mutation could decrease the migration of SH-SY5Y cells via the downregulating of RHOA and ROC.^49^ Meanwhile, during fetal development, *ARVCF* expression is closely associated with the rearrangement and migration of neurons within ganglionic eminence, a telencephalic structure which gives rise to precursor neurons of the striatum, amygdala and basal nucleus of Meynert.^50^ By using *Arvcf*-KO mice generated from this work, we found a decreased spatial hippocampus-dependent learning and memory. Furthermore, *Arvcf*-KO in mouse hippocampal CA3 also presented impaired learning and memory. These findings strongly indicated a specific action of *ARVCF* on hippocampus-dependent learning and memory in animals.

*ARVCF* is located on chromosome 22q11.2, one of the most significant regions (22q11.23–12.1) from linkage studies on ND,^55^ and is adjacent to *COMT* gene, a common target for genetic and functional researches of ND and other psychiatric disorders as well.^56^^,^^57^^,^^58^ Although an association of *ARVCF* with schizophrenia has been previously reported, whether this gene is associated with other addictive or psychiatric disorders has not been reported yet. Thus, this study represents the first one showing the presence of significant association of SNP rs148582811 in *ARVCF* with ND.

Given its genomic location and biological function in learning and memory, together with the existence of the functional SNP, we further investigated the biological effect of *ARVCF* on nicotine dependence. We found the wild-type mice presented a robust preference for nicotine in CPP test, but *Arvcf* knockout mice did not show any obvious preference, indicating that *Arvcf* plays a key role in mediating the rewarding effect of nicotine. Further, since nicotine is known to has an effect on hippocampus-dependent learning and memory^27^^,^^33^ except for the well-known rewarding effects, we also examined the function of *ARVCF* on learning and memory in nicotine chronically treated mice. We found that chronic nicotine administration impaired spatial and emotional memory which were consistent with previous findings.^59^^,^^60^ However, these hippocampus-dependent learning and memory deficits were abolished in *Arvcf* ^KO/KO^ mice. Taken together, these findings strongly indicate that *ARVCF* plays a critical role in nicotine-associated learning and memory.

### Conclusions

Our GWAS analysis discovered that SNP rs14858281 in *ARVCF* was significantly associated with ND and such an association was further validated not only in an independent sample from our sample collection at the SNP level, but also in the GTEx dataset at the gene level. Moreover, our RNA-seq analysis of mRNA expression in both our own sample and public GEO dataset revealed that *ARVCF* is lowly expressed in smokers and EMIC analysis of the *ARVCF* expression data in the forebrain region of GTEx dataset revealed that *ARVCF* is a causal gene of ND and *ARVCF* overexpression might increase the risk of ND. Next, our *in vitro* reporter assay indicated that the SNP rs14858281 regulates *ARVCF* expression in an allele-specific manner, which is realized by binding to the transcriptional factor XRCC5. Finally, by generating *Arvcf*-KO mice, we demonstrated that *Arvcf* deletion impaired hippocampus-dependent learning and memory and alleviated nicotine-induced memory deficits. Taken together, this study demonstrated for the first time that SNP rs14858281 is significantly associated with ND and *ARVCF* plays a key role in nicotine-associated hippocampus-dependent memory.

### Limitation of the study

There were several limitations of this study that should be noted. Firstly, we did not perform the validation of our genetic findings in other ethnic populations, in which the genetics of smoking behaviors differ from each other.^61^^,^^62^ Secondly, as our genetic and functional data have confirmed the involvement of rs148582811 and the DNA region around it in learning, memory and ND as well, more effort is needed to find causal variant(s) in this region and *ARVCF* gene through deep sequencing analysis. Thirdly, although *ARVCF* plays an important role in memory formation and nicotine-induced memory deficits, there was a lack of evidence to clarify the molecular mechanisms of *ARVCF* in the process of memory and rewarding effects of nicotine, which is what we plan to do in future research.

## STAR★Methods

### Key resources table

**Table:** 

REAGENT or RESOURCE	SOURCE	IDENTIFIER
**Antibodies**		

anti-ARVCF	Santa Cruz	sc-14827
anti-XRCC5	Proteintech	66546-1-Ig
anti-β-actin	Abcam	ab8227
Goat anti-rabbit IgG	Abcam	ab6721
Goat anti-mouse IgG	Abcam	ab6789

**Bacterial and virus strains**		

AAV8-shArvcf-eGFP	OBiO Technology	N/A
Human blood sample	Participants were recruited from local communities in the city of Jincheng, Shanxi Province, China	N/A

**Chemicals, peptides, and recombinant proteins**		

Nicotine	Sigma-Aldrich	N3876
Lipofectamine 2000	Invitrogen	11668019
Trizol	Life Technologies	15596026
RIPA	Beyotime	P0013C

**Critical commercial assays**		

QuikChange Lightning Multi Site-Directed Mutagenesis Kit	Agilent	210513
sgRNA-PX459 by Viromer® RED	Lipacolyx	VR-01LB-01
QIAmp DNA Mini Kit	QIAGEN	51304
NEBNext® Ultra™ DNA Library Prep kit	Illumina	E7103-E7645
iScript cDNA Synthesis kit	Bio-Rad	1708891
Dual-Lucifearse Reporter Assay	Promega	E1910
BCA protein assay	Beyotime	P0010
NE-PER™ Nuclear and Cytoplasmic Extraction Reagents kit	Thermo Fisher Scientific	78833
LightShift Chemiluminescent Kit	Thermo Fisher Scientific	20148

**Deposited data**		

OMRF Microarray Core Facility Human Print	https://ncbi.nlm.nih.gov	GDS1348
RNA Sequencing Data	https://bigd.big.ac.cn/gsa/	CRA011029
GTEx eQTL dataset	http://www.ncbi.nlm.nih.gov/projects/gap/eqtl/index.cgi?	GTEx eQTL (v8)

**Experimental models: Cell lines**		

Human: neuroblastoma cell line SH-SY5Y	This study	N/A
Human: embryonic kidney cell line, HEK293T	This study	N/A

**Experimental models: Organisms/strains**		

Mouse: C57BL/6	GemPharmatech	N/A
ARVCF knockout mice	GemPharmatech	N/A

**Oligonucleotides**		

Target region PCR, see Table S2	This study	N/A
Primers for XRCC5: Forward primer (5′- CGCAAATGGGCGGTAGGCGTGT-3′); Reverse primer (5′-TAGAAGGCACAGTCGAGG-3′)	This study	N/A
sgRNAs targeting rs148582811-containing DNA region, see Table S2	This study	N/A
qPCR primers, see Table S2	This study	N/A

**Recombinant DNA**		

pRL-TK	Promega	E2312
pGL4.26	Promega	E8441
pcDNA3.1	Invitrogen	V80020
XRCC5-siRNA	GenePharma	N/A
Plasmid: PX459	Addgene	Plasmid ID: 62988

**Software and algorithms**		

Samtools	Li et al.^63^	http://samtools.sourceforge.net/
GATK	McKenna et al.^64^	https://gatk.broadinstitute.org/hc/en-us
BEAGLE (v. 4.1)	Browning et al.^65^	https://faculty.washington.edu/browning/beagle/b4_1.html
PLINK (v. 1.07)	Purcell et al.^66^	https://zzz.bwh.harvard.edu/plink/
GATES	Li et al.^67^	N/A
EMIC on KGGSEE	Jiang et al.^68^	https://pmg-lab-docs.readthedocs.io/en/latest/KGGSEE_doc/KGGSEE.html#gene-based-causality-analysis
Water Maze 4.07	Actimetrics	N/A
FreezeFrame 3	Coulbourn Instruments	N/A
GraphPad Prism 8.0	GraphPad Software lnc	https://www.graphpad.com/

**Other**		

Mini-osmotic pumps	Alzet	Model 1002

### Resource availability

#### Lead contact

Further information and requests for resources and reagents should be directed to and will be fulfilled by the lead contact, Dr. Ming D. Li (ml2km@zju.edu.cn).

#### Materials availability

This study did not generate new unique reagents. All chemicals were obtained from commercial resources and used as received.

#### Data and code availability

• All data reported in this paper are publicly available as of the date of publication. Accession numbers are listed in the key resources table.
• This paper does not report any original code.
• Any additional information required to reanalyze the data reported in this work paper is available from the lead contact upon request.

### Experimental model and study participant details

#### Study subjects

All included Han Chinese participants were from local communities in the cities of Jincheng and Taiyuan, Shanxi Province during 2012-2014.^69^^,^^70^ Because few females (∼4%) smoke tobacco in China, only males (n = 5055) were included in the study. The participants who were 20 years or older were recruited and interviewed by experienced researchers. Smoking status and other demographic characteristics, such as age, gender, education, marital status, and medical history, were all collected from each participant. Subjects who were diagnosed by DSM-IV as psychiatric disorders such as schizophrenia and major depressive disorders were excluded from the study. A total of 5,055 biologically unrelated subjects were included for the study, with 1,329 subjects used for the first phase of whole genome-sequencing analysis (805 smokers and 524 age-matched non-smokers) and 3,726 subjects used for the validation phase (1,811 smokers and 1,915 non-smokers). All materials related to the study were approved by the Institutional Review Board of the First Affiliated Hospital of Zhejiang University School of Medicine, and the written informed consent was obtained from all participants (Approval #: 2011-04).

#### Generation of plasmid constructs

The DNA fragment containing the C allele of rs148582811 overlapped with H3K4me1 and H3K27ac peaks was amplified by using genomic DNA as template with the following primers: forward: 5′-CCTCACCACCCAGCTTAGAG-3′, and reverse: 5′-GAAAAAGGGCCCCCTAGTGA-3′. The amplified PCR product was subsequently cloned into pGL4.26 vector (Promega). The T allele of rs148582811 was introduced by using QuikChange Lightning Multi Site-Directed Mutagenesis Kit (Agilent) according to the manufacturer’s instructions. The X-ray repair cross-complementing protein 5 (XRCC5) cDNA was amplified by using the forward primer (5′-CGCAAATGGGCGGTAGGCGTGT-3′) and reverse primer (5′-TAGAAGGCACAGTCGAGG-3′), respectively, which was then cloned into a pcDNA3.1 expression vector (Invitrogen). Single guide RNAs (sgRNAs) targeting rs148582811-containing DNA region were designed by the CRISPR Design Tool (http://tools.genome-engineering.org/) (Table S2) and cloned into PX459 plasmid (Addgene plasmid ID: 62988). All constructed plasmids were verified by Sanger sequencing (Sangon Biotech).

#### Genome editing in HEK293T and SH-SY5Y cells

Both HEK293T and SH-SY5Y cells, cultured on 24-well plates at 80%-90% confluency, were transfected with sgRNA-PX459 by Viromer® RED (Lipacolyx). For rs148582811 knockout (rs148582811-KO) editing, sgRNA-KO-1 and sgRNA-KO-2 constructs were co-transfected into cells. For SNP rs148582811-T editing, the sgRNA-T construct was co-transfected with homologous repair template with rs148582811-T allele. The medium for cell culturing was changed 6 h after transfection. After 24 h of transfection, puromycin was added to select transfected positive cells. The genomic DNA from transfected positive cells was extracted by QIAmp DNA Mini Kit (QIAGEN), in which the target region was amplified by PCR and sequenced by Sanger sequencing (Sangon Biotech).

#### Generation of Arvcf knockout animals

The wild-type (WT) mice were purchased from GemPharmatech Co. Ltd (Nanjing, China). The *Arvcf* ^WT/KO^ and *Arvcf* ^KO/KO^ mice were generated via the CRISPR/Cas9 system at GemPharmatech Co. Ltd (Nanjing, China). All animals were maintained in the Animal Core Facility at Zhejiang University. All mice were group-housed (3-5 per cage) under a 12 h light-dark cycle with food and water *ad libitum*. Mice were 8-10 weeks of age at the time of conducting experiments. In various behavioral experiments, the ratio of male and female mice was 1:1. The project was approved by the Animal Care and Use Committee of the First Affiliated Hospital of Zhejiang University (Approval #: 2023-596).

### Method details

#### Whole genome sequencing

Genomic DNA was extracted from peripheral blood samples using Blood DNA isolation kit (QIAGEN). Sequencing libraries were generated using NEBNext® Ultra™ DNA Library Prep kit for Illumina (NEB, USA) according to the manufacturer’s recommendations and were sequenced to produce 150-bp paired-end reads on an Illumina HiSeq2000 platform. After filtering out reads with poor-qualities, they were aligned to NCBI Build 37 (hg19) of the human reference sequence using BWA-MEM with default parameters.^71^ SAMtools was applied to index the alignments in BAM format,^63^ and Picard (v. 2.5.0) was used to detect PCR duplicate reads. Only non-duplicate reads were used for the downstream analysis. The generated BAM files were then realigned and recalibrated using GATK.^64^

#### Whole genome variant calling

Multi-sample variant calling was performed on all BAM files with GATK HaplotypeCaller. The option of ‘--genotype_likelihood_model’ was set to ‘BOTH’ with the use of default annotation outputs for variant calling. By using the ‘--dbSNP’ option, dbSNP (v. 147) was applied to fill in the variant ID column of the result variant call format (VCF) files. All of the HaplotypeCaller calling processes were conducted with the pipeline of bcbio-nextgen (v. 0.9.9). After initial variant calling, we applied a series of variant filters to obtain a high confidence set of variants. Finally, we obtained a total of 32,482,876 single nucleotide variants (SNVs) from autosomes (Table S1) for genotype refinements by using BEAGLE (v. 4.1).^65^

#### Single-point association analysis

The genome-wide single-point association analysis was performed with PLINK (v. 1.07)^66^ under an additive genetic model for each ND measure. The standard quality control criteria for sequenced data included: SNP and sample call rate ≥ 99%, Hardy-Weinberg Equilibrium (HWE) < 10^-6^, and minor allele counts (MAC)≥ 30. Population stratification was evaluated via the multidimensional scaling (MDS) implemented in PLINK (v. 1.07). The first three MDS components, age, and gender were adjusted in the genome-wide association analysis. A P-value of 5 × 10^-8^ was defined as the threshold for genome-wide significance. Combined with other replication samples, we performed a meta-analysis using the inverse variance method implemented in the computer program METAL.^72^

#### Gene-based association test using extended Simes procedure (GATES)

We used GATES procedure to determine significance of *ARVCF* with ND by using our GWAS summary statics through “--gene-assoc” in KGGSEE.^67^ We included all SNPs with a MAF of 0.01 in the analysis if they were located within the 5 kb from either the upstream or downstream of the gene. LD scores for all included SNPs were calculated by using the 1000 Genomes Project Phase 3 of East Asian sample reference genotypes (hg19). By using the same approaches in KGGSEE with the specified eQTL summary statistics, we also did gene-based association test for *ARVCF* based on the GTEx eQTLs data.

#### The effective-median-based Mendelian randomization for inferring the causal genes (EMIC) analysis

We employed EMIC analysis in KGGSEE to infer the causal effect of *ARVCF* expression on ND phenotype.^68^ We calculated GWAS p-values of all variants within 1 MB upstream and downstream of *ARVCF* and used the pre-calculated eQTLs (p < 0.01) results in 13 brain tissues of GTEx (v8),^35^ by filtering out those variants with a MAF of less than 0.01 and HWD test p-value of greater than 1.0E-6. The 1000 Genomes Project Phase 3 of East Asian sample reference genotypes (hg19) were used to calculate LD score for all included SNPs. The SNPs that affect both phenotype and gene expression are considered instrumental variables (IV).

#### Technical and independent validations

To validate the accuracy of identified SNPs from WGS, we selected 256 common SNPs including those top-ranked SNPs from the abovementioned GWAS for genotyping by using *TaqMan* OpenArray genotyping platform (Applied Biosystems) as we reported previously.^69^^,^^73^^,^^74^^,^^75^ The concordance between sequencing and genotyping was assessed by using Pearson correlation (Figure S2). A total of 3,726 unrelated Chinese participants consisting of smokers (N = 1,811) and non-smokers (N = 1,915) were used for validation phase, which were completely independent from the subjects used for WGS analysis in the discovery stage.

#### Luciferase reporter assay

A total of 1.3 × 10^5^ HEK293T cells were seeded onto 24-well plates one day prior to transfection. The luciferase reporter constructs (Promega) were co-transfected with pRL-TK vector (Promega) by Lipofectamine 2000 (Invitrogen). To assess the effect of XRCC5 on transcriptional activity, luciferase reporter construct was transfected with XRCC5-siRNA (GenePharma). After 48 h of transfection, cells were lysed and assayed with the Dual-Lucifearse Reporter Assay System (Promega) according to the manufacturer’s instructions. In all experiments, the *Firefly* luciferase activity was normalized to the *Renilla* signal and reported as proportions relative to the control samples.

#### Quantitative RT-PCR analysis (qRT-PCR)

Total RNA was extracted from SH-SY5Y or HEK293T cells using Trizol reagent (Life Technologies). The RNA quality and quantity were measured with a NanoDrop ND2000 Spectrophotometer (Thermo Scientific). Reverse transcription was performed by iScript cDNA Synthesis kit (Bio-Rad) using random primers and 1 μg total RNA. The mRNA expression level of *ARVCF* and *XRCC5* was determined by qRT-PCR under the following the conditions: 50°C for 2 min, 95°C for 10 min, and 40 cycles of 95°C for 15 sec and 60°C for 1 min. The qRT-PCR data was analyzed by the comparative 2^-ΔΔCt^ method^76^ with Glyceraldehyde 3-phosphate dehydrogenase (*GAPDH*) used as a reference control.

#### Western blotting

Proteins from cells or tissues were extracted using RIPA buffer (Beyotime) containing 1 mM phenylmethylsulfonyl fluoride (Beyotime) and quantified using the BCA protein assay (Beyotime). Proteins were electrophoresed on 8% sodium dodecyl sulfate-polyacrylamide gel (Beyotime) and transferred onto PVDF membrane (Millipore). After blocked in Tris-Buffered Saline with Tween (TBST) containing 5% albumin bovine (BSA) (Amresco), membranes were incubated with primary antibody at 4°C overnight followed by secondary antibody at room temperature for 1 h. The primary antibody used for Western blotting was anti-ARVCF antibody (1:1000; Santa Cruz Biotechnology), anti-XRCC5 (1:1000; Proteintech) and anti-β-actin (1:5000; Abcam). The secondary antibody used was horseradish peroxidase-conjugated goat anti-rabbit IgG and anti-mouse IgG (1:5000; Abcam). Antibody-bound proteins were visualized by incubating with Clarity Western ECL substrate (Bio-Rad) for 2 min. The immunoblot results were quantified by using ImageJ software (National Institutes of Health) and then normalized to β-actin.

#### Electrophoretic mobility shift assay (EMSA)

Nuclear extracts were prepared from SH-SY5Y cells by using the NE-PER™ Nuclear and Cytoplasmic Extraction Reagents kit (Thermo Fisher Scientific) according to manufacturer’s instructions. The biotin-labeled probes containing SNP rs148582811 T or C allele were synthesized by Sangon Biotech. The sequences of the probe with C allele are: 5’-AGCCCCATGGGCTCTCCTCACCTGGCTGGGGCATGTGGGG-3’ (forward), and reverse: 5’-CCCCACATGCCCCAGCCAGGTGAGGAGAGCCCATGGGGCT-3’. The sequences of the probe with T allele are: 5’-AGCCCCATGGGCTCTCCTCATCTGGCTGGGGCATGTGGGG-3’ (forward), and 5’-CCCCACATGCCCCAGCCAGATGAGGAGAGCCCATGGGGCT-3’ (reverse). Unlabeled probes were also synthesized with the same sequences for specific competition of labeled probes. The binding reaction was performed with LightShift Chemiluminescent Kit (Thermo Fisher Scientific). For each binding reaction, 5 μg nuclear protein extracts were incubated with 1× binding buffer, 2.5% glycerol, 5 mM MgCl_2_, 50 ng/μl Poly (dI⋅dC), 0.05% NP-40, and 20 fmol of biotin-labeled probes at room temperature for 30 min. For competition assay, 4 pmol unlabeled probes were preincubated for 5 min at room temperature with nuclear extracts before the addition of the labeled probes. For super-shift assay, nuclear extracts were preincubated with 10 μg XRCC5 (Proteintech) antibody on ice for 30 min. The DNA-protein complexes were separated on a 6% non-denaturing polyacrylamide gel by using 0.5× Tris-borate-EDTA (TBE) buffer, and then transferred to nylon membrane on ice. The DNA was then crosslinked to the membrane by UV cross-linker. The signal of the biotin-labeled DNA was detected with a LightShift Chemiluminescent EMSA kit.

#### DNA pull-down assay

For each binding reaction, the biotin-DNA used in EMSA was incubated with streptavidin-agarose beads (Sangon biotech) in the binding buffer consisting of 60 mM KCl, 12 mM HEPES (pH 7.9), 25 mM Tris-HCl (pH 7.5), 1 mM EDTA, 5% glycerol, 100 mM NaCl, 1 mM DTT, 10 ng/μl Poly(dI⋅dC) and 0.5% protease inhibitors cocktail at room temperature for 30 min. Following incubation, the beads were washed thoroughly with binding buffer to eliminate excess DNA. Next, DNA-coated beads were incubated with nuclear extracts prepared from SH-SY5Y cells in binding buffer at 4°C overnight, and then washed three times to remove unbound proteins. Beads-bound proteins were boiled in 5× loading buffer and subjected to polyacrylamide gel electrophoresis followed by silver staining and mass spectrometry analysis. The eluted proteins from the beads without the biotinylated DNA probe were used as control.

#### Viral injection

The virus AAV8-sh*Arvcf*-eGFP (1.09 × 10^13^ particles/ml) was purchased from OBiO Technology. Mice were anesthetized with sodium pentobarbital (1% wt/vol) via an intraperitoneal injection. For *Arvcf* knockdown, 0.3 μl AAV8-sh*Arvcf*-eGFP was injected bilaterally into hippocampus CA3 region (anteroposterior AP: -2.46; mediolateral ML: ±3.1; dorsoventral DV: -2.9; mm relative to bregma) using stereotaxic equipment (RWD Life Science). Three weeks after injection, various behavioral tests (see below for details) were performed on these animals.

#### Nicotine conditioned place preference (CPP) study

The CPP apparatus consisted of two chambers, divided by a central neutral area, one chamber had white walls with a gridded floor and the other had dark walls with a hole floor. Removable partition can isolate mice within the chambers, and their removal allowed mice to move freely between the two chambers. On the first day, mice entered from the central area and were allowed to move freely in the two chambers for 15 min, the time spent in each chamber was recorded and used to determine the natural preference of the mice for the chamber context. The non-preferred side of mice with less residence time was used as the drug-paired side chamber. Both WT and *Arvcf* ^KO/KO^ mice were randomly assigned to the two experimental groups for conditioning session on days 2-8. Nicotine conditioned place preference in mice were measured by subcutaneous injection at a dose of 0.5 mg/kg/day per mouse. Nicotine (Sigma-Aldrich) were dissolved in 0.9% NaCl (pH 7.0). During conditioning sessions, mice were confined to the drug-paired side chambers for 30 min after nicotine or saline injection. Five hours later, mice were confined to the opposite compartment for 30 min after saline injection. On the test day (day 9), mice were allowed to enter all chambers for 15 min in a drug-free state and the time spent in each chamber was recorded. The mouse behavioral activity was captured with an overhead camera. The preference score was calculated as the difference between the time spent on the drug-paired side and the opposite side of chamber. A positive value reflects a conditioned preference for the drug-paired side and a negative value reflects conditioned place aversion.

#### Chronic nicotine administration

Both WT and *Arvcf* ^KO/KO^ mice were administrated with saline or nicotine by using mini-osmotic pumps (Model 1002, Alzet) for 14 days. In the present study, the dosage of nicotine was 6.3 mg/kg/day based on previous studies.^18^^,^^60^^,^^77^ Each pump was implanted on the back of mouse under isoflurane anesthetic. The Morris water maze (MWM) test was performed during days 8-13 after pump implanted, followed by the fear conditioning test on day 13-14.

#### Novel object recognition (NOR) test

The NOR test was performed in the familiarization stage, each mouse was placed in an open-field arena (45 × 45 × 45 cm^3^) containing two identical objects for 8 min.^78^ Twenty-four hours after familiarization, each mouse was placed back in arena with one familiar object and one novel object for 5 min. Before each test, the apparatus and objects were cleaned with 75% ethanol solution. Every animal’s tracks were monitored with ANY-maze video-tracking software. Object recognition memory was analyzed by dividing the amount of exploring time of the mouse spent on the novel object over total object exploration time (namely, discrimination ratio). Object exploration was defined as duration of the mouse’s head toward the object within 2 cm or its nose touching the object.

#### Morris water maze (MWM) test

The MWM test was performed by using a water pool (21°C-23°C, 24 cm deep, 100 cm diameter) that was clouded with nontoxic titanium dioxide. The pool was divided into four virtual quadrants. A circular platform, 10 cm in diameter, was fixed in one of the quadrants 1 cm below the water surface. Mice were subjected to four training sessions per day for 5 days and a memory retention test (probe test) on the sixth day. In the training session, mice were placed at the same starting position in each quadrant and allowed them to swim freely to find the hidden platform for 60 s. If the mouse failed to locate the platform within 60 s, it was guided to the platform and allowed to stay on the platform for 10 s. On the sixth day, the platform was removed, and mice were allowed to swim freely for 60 s. The movements were recorded with a camera directly above of the pool. The swimming speed, distance travelled, time to reach the previous platform location, the number of times crossing the original platform site were all collected and analyzed by Water Maze 4.07 software (Actimetrics, Wilmette, IL, USA).

#### Fear conditioning test

In this test, two environmental contexts were applied. Context A was a square chamber with an electrical grid floor for providing foot-shocks to animals. Before each behavioral assessment, 75% ethanol solution was used to clean chamber. Context B was a cylindrical chamber with a flat floor, and the texture of the wall was a black-and-white square mosaic. This chamber was cleaned with 1% acetic acid prior to start each trial. On the first day, animals were placed individually into the context A for conditioning, in which three tones (30 s, 5 kHz, 85 dB) were presented and co-terminated with a foot shock (0.8 mA, 2 s). Tone-shock pairings began after 120 s delay and were separated by interstimulus interval. To examine contextual fear memory, each mouse was returned to context A after 24 h. No shock or tone was presented during the 5 min session. After 2 h of the contextual test, the cued fear memory was tested by exposing each mouse to a novel context B for 3 min, followed by presentation of 3 min tone. The FreezeFrame 3 software (Coulbourn Instruments) was used to control auditory and electrical stimuli as well as calculate freezing response. The percentage of freezing time was calculated during each test.

#### Immunofluorescence staining

Mice were deeply anesthetized and perfused with cold saline followed by 4% paraformaldehyde (Sangon Biotech). Brains were dissected and post-fixed in 4% paraformaldehyde at 4°C overnight and then transferred to 30% sucrose in 0.1 M PBS (pH 7.4). Coronal sections (50 μm) were collected serially using a freezing microtome (Leica CM3050S) and stored in 0.1 M PBS. The sections were incubated in blocking buffer containing 5% normal goat serum in 0.2% Triton X-100/PBS (PBST) for 1 h at room temperature and then with primary antibody overnight at 4°C. After three washes with PBST, sections were incubated with Alexa Fluor 633-conjugated secondary antibody for 1 h at room temperature. Nuclear staining was performed using DAPI and then sections were mounted on slides. Immunofluorescence was assessed by using a laser confocal microscope (Nikon).

### Quantification and statistical analysis

The GraphPad Prism 8.0 software was used for statistical analysis. All data were presented as Mean ± Standard Error of the Mean (SEM). Two-tailed unpaired t-test was used to perform statistical comparisons between two groups. One-way analysis of variance (ANOVA) followed by Tukey's multiple comparison test was performed for the comparisons among more than two groups. The statistically significance was determined at p < 0.05. ∗p < 0.05; ∗∗p < 0.01; ∗∗∗p < 0.001; ∗∗∗∗p < 0.0001; ns = no significance.
