# Supplementary material for: Single nucleotide polymorphisms rs148582811 regulates its host gene ARVCF expression to affect nicotine-associated hippocampus-dependent memory
Source: iScience. 2023 Oct 28;26(12):108335. doi: 10.1016/j.isci.2023.108335 (PMC10679859; doi:10.1016/j.isci.2023.108335)
Supplement: Document S1. Figures S1–S4 and Tables S1–S3 [file mmc1.pdf]

iScience, Volume *26*

Supplemental information

Single nucleotide polymorphisms rs148582811 regulates its host gene *ARVCF* expression to affect nicotine-associated hippocampus-dependent memory

Zhongli Yang, Jiali Chen, Haijun Han, Yan Wang, Xiaoqiang Shi, Bin Zhang, Ying Mao, Andria N. Li, Wenji Yuan, Jianhua Yao, and Ming D. Li

SUPPLEMENTAL INFORMATION

Table S1. Variant information of current WGS study (*Related to STAR Methods*)

SNVs	High-sensitive SNVs	High-confidence SNVs
No. of SNVs	35,052,791	32,482,876
No. of known variants	16,740,973	15,709,117
No. of novel variants	18,311,818	16,773,759
Novelty rate (%)	52%	62%
Transition/Transversion ratio	1.99	2.12
Common variants (MAF $\geq$ 5%)	6,148,144	5,681,945
Rare variants (MAF < 5%)	28,900,829	26,806,315

Note: SNVs = Single nucleotide variants，MAF = Minor Allele Frequency.

Table S2. Primer sequences used in this study (*Related to STAR Methods*)

Purposes	Forward (5'-3')	Reverse (5'-3')
sgRNA-KO-1	CACCGTGACTCTGGGGACCTGCGCC	AAACGGCGCAGGTCCCCAGAGTCAC
sgRNA-KO-2	CACCGCTGCTGGCCGACACCAAGTC	AAACGACTTGGTGTCGGCCAGCAGC
sgRNA-T	CACCGGGGCTCTCCTCACCTGGCTG	AAACCAGCCAGGTGAGGAGAGCCCC
Template-T	GAGAAAGGGAGCAAACGCTGGTCTCTGTTGAGGCAGCCCC ATGGGCTCTCCTCATCTGGCTGGGGCATGTGGGGCTCAGA	
Target region PCR	CCTCACCACCCAGCTTAGAG	GAAAAAGGGCCCCCTAGTGA
For qPCR:		
ARVCF	TGACAACAAGTCGGTGGAGAAC	GTGCACGTGGTAGGACAGGTT
XRCC5	GGACGTGGGCTTTACCATGA	AGCAAACACCTGTCGCTGTA
GAPDH	CGACAGTCAGCCGCATCTT	CCGTTGACTCCGACCTTCA

Table S3. A list of 31 SNPs identified in this WGS study (*Related to Table 2*)

CHR	Gene	SNP	WGS_P	WGS_MAF	WGS_Phenotype
2	NA	rs796305983	2.19E-11	0.05342	Smoking
3	*NAALADL2*	rs62287013	1.71E-10	0.01317	CPD
18	*ZNF516*	rs150835374	2.84E-09	0.01129	CPD
22	*DGCR6-PRODH*	rs796774020	3.20E-09	0.1005	Smoking
2	42kb 3' of *ACSL3*	rs147450867	4.00E-09	0.01242	CPD
22	*RBFOX2*	rs148758910	7.35E-09	0.01204	CPD_Index
1	40kb 5' of *ATP2B4*	rs147489854	1.18E-08	0.01693	CPD_Index
17	*RP11-260A9.6*	rs9674567	1.21E-08	0.03574	Smoking
1	*SRGAP2B*	rs546609	1.22E-08	0.02107	Smoking
1	*PLD5*	rs2654869	1.24E-08	0.01016	CPD_Index
1	*OR6N2*	rs12027473	1.49E-08	0.0617	CPD
12	*RPH3A*	rs41473045	1.49E-08	0.03348	CPD
13	*INTS6*	rs76543138	1.51E-08	0.05794	CPD
1	*NBPF12*	rs58278369	1.71E-08	0.1144	Smoking
10	*WARS2P1*	rs144941005	1.81E-08	0.02859	CPD
21	*AGPAT3*	rs73373146	1.85E-08	0.01242	CPD

1	*PFN1P2*	rs2940057	1.96E-08	0.07901	Smoking
2	*COL4A3*	rs72975987	2.12E-08	0.1975	ND
14	*IFI27,IFI27L2,IFI27L1*	rs185177063	2.58E-08	0.0158	CPD
10	*CTBP2*	rs3208623	3.19E-08	0.0696	Smoking
7	*CACNA2D1*	rs144256292	3.28E-08	0.01242	CPD
3	150Kb 3' of *FOXP1*	rs7635815	4.86E-08	0.02295	CPD
18	65kb 3' of *ZNF519*	rs1073861	6.65E-08	0.1843	CPD
3	169kb 5' of *U6*	rs62237500	8.52E-08	0.03499	CPD
1	*LPPR4*	rs78343536	9.40E-08	0.06	CPD
20	*Y_RNA*	rs73613165	9.72E-08	0.1584	Smoking
22	*ARVCF*	rs148582811	1.18E-07	0.01166	CPD
1	*DRD5P2*	rs2907220	1.68E-07	0.193	Smoking
12	*FGD6*	rs7485352	2.20E-07	0.09443	FTND
1	*LMO4*	rs79092926	2.81E-07	0.04778	Smoking
1	*PLXNA2*	rs10158303	7.25E-07	0.02107	FTND

Note: WGS_P = WGS_P -value，CHR = Chromosome，MAF = Minor Allele Frequency, CPD = Cigarettes smoked per day.

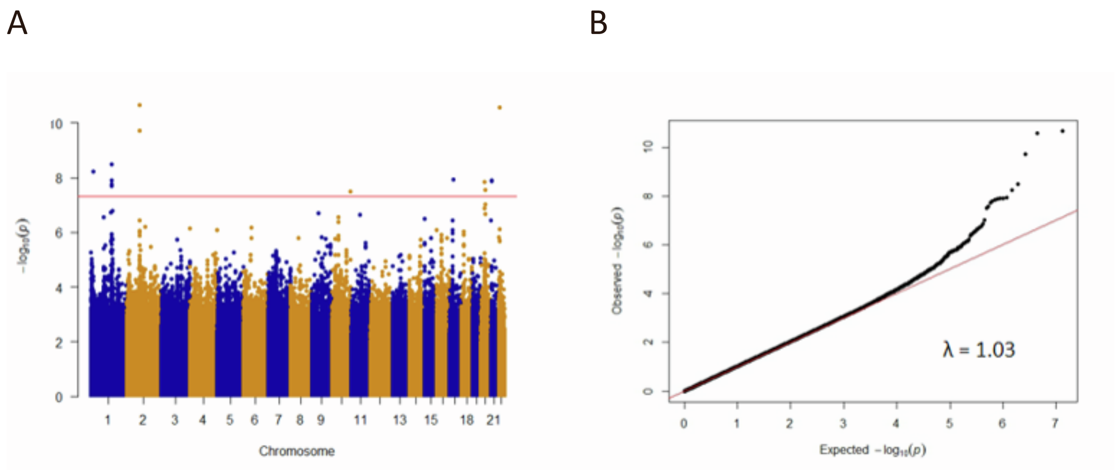

Figure S1. Genome-wide association analysis results. (A) Manhattan plot for smoking status (*Related to Table 1*). Red dashed lines represent significant threshold for WGS analysis (5.0×10^{-8}). (B) Quantile-quantile plot for smoking status (Lambda = 0.974).

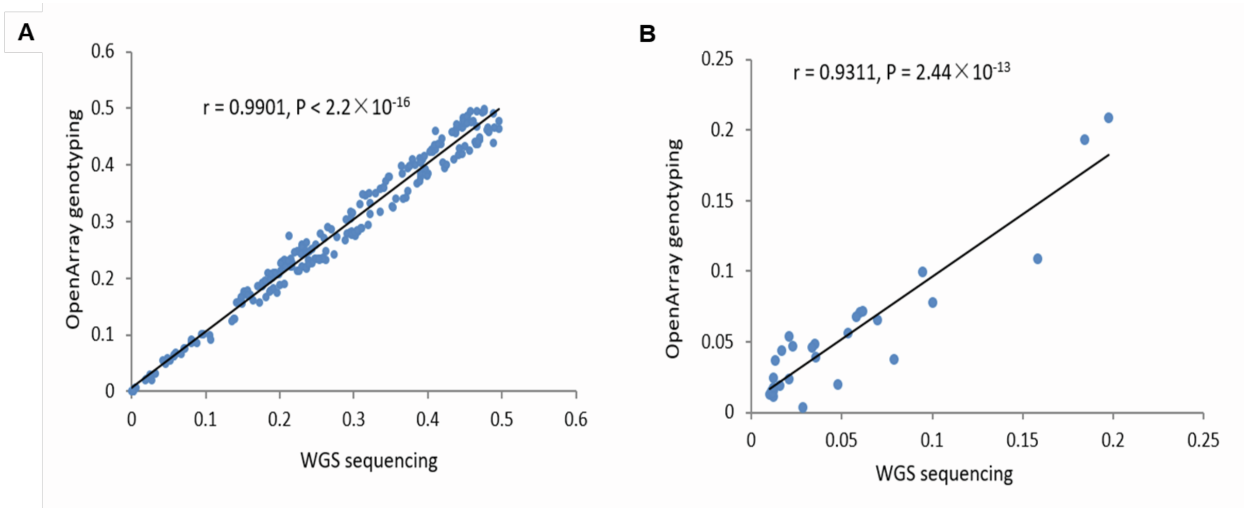

Figure S2. The concordance between variants in WGS study and that in OpenArray genotyping (*Related to STAR Methods*). (A) The concordance between selected 256 SNPs from OpenArray genotyping and WGS study. **(B)** The top-ranked SNPs identified from WGS study and OpenArray genotyping.

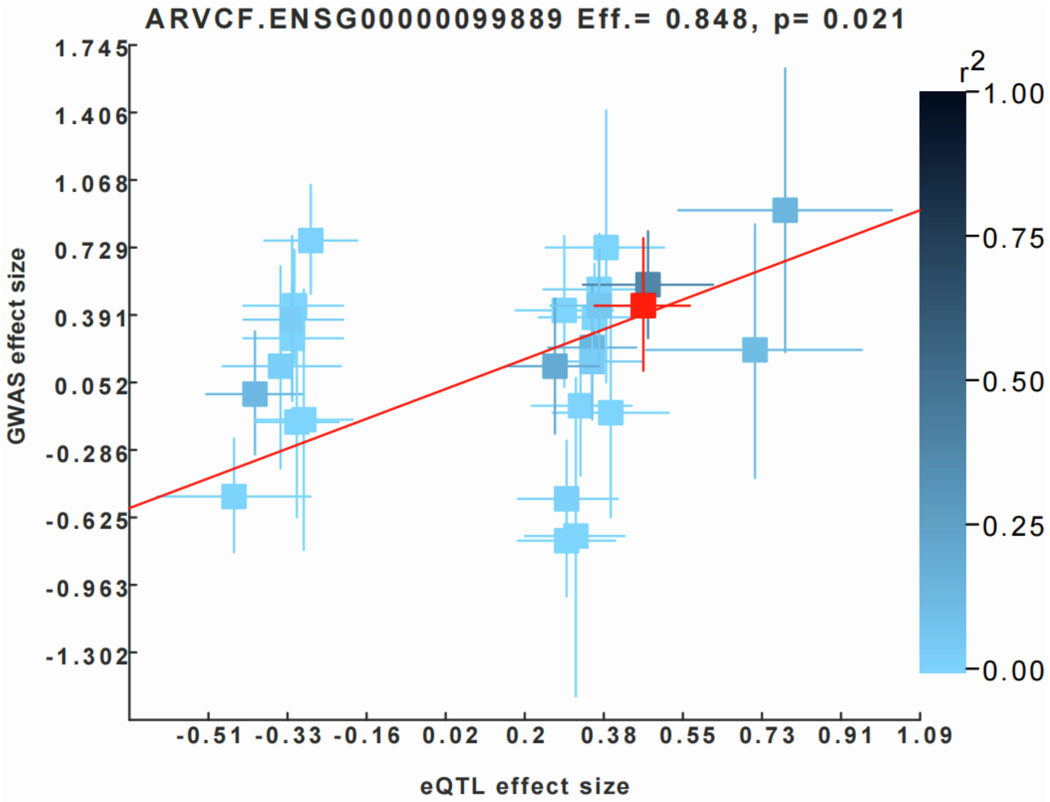

Figure S3. Scatter plot of genetic association with *ARVCF* expression for ND by using the eQTL results in the forebrain region of GTEx dataset (*Related to Figure 1*). A filled rectangle on the plot denotes an instrumental variable (IV). The red rectangle denotes the most significant GWAS variant among all of the IVs within the *ARVCF* gene. The slope of the black line is the estimated causal effect by EMIC. The gray level of an IV denotes the degree of the LD between the IV and the most significant GWAS variant. The error bars in the rectangles denote the standard errors of the coefficient estimates.

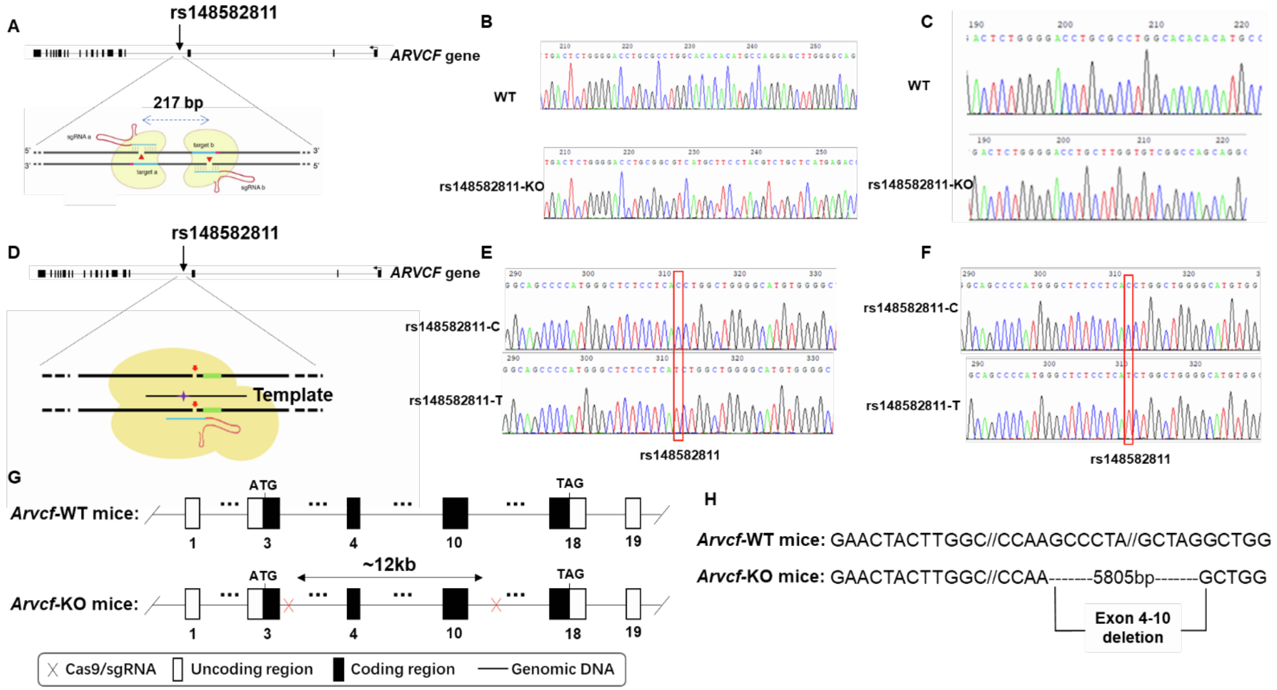

Figure S4. Schematic graphs to construct rs148582811 knockout (rs148582811-KO) and rs148582811-T cell lines, as well as *Arvcf* knockout (*Arvcf*-KO) mice by applying CRISPR/Cas9 system (*Related to STAR Methods*). (A) Schematic strategy of sgRNA-targeting site in human ARVCF gene for rs148582811-KO. Sanger sequencing results of construction rs148582811-KO in SH-SY5Y **(B)** and HEK293T **(C)** cell lines. **(D)** Schematic of rs148582811-T editing strategy by introducing a template. Sanger sequencing results of construction rs148582811-T in SH-SY5Y **(E)** and HEK293T **(F)** cell lines. **(G)** Strategy of constructing *Arvcf*-KO mice by deleting exons 4-10. Abbreviations: ATG = start codon, TAG = stop codon. (**H**) Sequences of *Arvcf* wild-type (*Arvcf*-WT) mice and *Arvcf*-KO mice by deleting 5805 bp among exons 4-10.
